# Early calcific degeneration of the St. Jude Medical Epic aortic bioprosthesis

**DOI:** 10.1002/ccr3.2664

**Published:** 2020-01-14

**Authors:** Mohammad Bashar Izzat, Nada Sabbagh, Hazem Aljasem

**Affiliations:** ^1^ Department of Surgery Faculty of Medicine Damascus University Damascus Syrian Arab Republic

**Keywords:** aortic, bioprosthesis, calcification, surgery, valve

## Abstract

This report highlights the need for close surveillance of bioprosthetic valves. Unaccountable degeneration of bioprosthetic valves can develop early after implantation and usually requires replacing the failed valve with a mechanical prosthesis.

## Graphical abstract

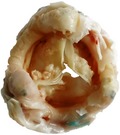

## 1 INTRODUCTION

Failure of bioprosthetic heart valves commonly occurs late after implantation and is typically due to gradual calcific degeneration of valve leaflets.^1^ In contrast, early deterioration of bioprosthetic valves is rare, and risk factors for this occurrence have not been completely uncovered.^2^

Here, we present an unusual case of early calcific degeneration of the Epic bioprosthetic valve (St. Jude Medical, St. Paul, MN) in the aortic position less than 2 years after implantation.

## 2 CASE REPORT

This is the case of a 69‐year‐old lady with normal renal function and who receives neither regular medications nor dietary supplements. This patient had undergone elective aortic valve replacement 20 months before for a severe degenerative aortic valve stenosis. A 19‐mm Epic bioprosthetic valve had been implanted due to the small aortic annulus, and immediate postoperative echocardiography had confirmed an estimated mean gradient across the bioprosthetic valve of 11 mm Hg and an effective valve orifice area of 1.6 cm^2^, with preserved left ventricular function. During postoperative follow‐up, 6‐monthly echocardiography documented progressively increasing gradients across the bioprosthetic valve and decreasing effective valve orifice areas, and the patient developed worsening shortness of breath. By 18 months after the operation, echocardiography showed critical stenosis of the bioprosthetic valve, with a mean gradient of 66 mm Hg and an effective valve orifice area of 0.80 cm^2^; hence, decision was made to replace the degenerative bioprosthetic valve electively.

**Figure 1:**
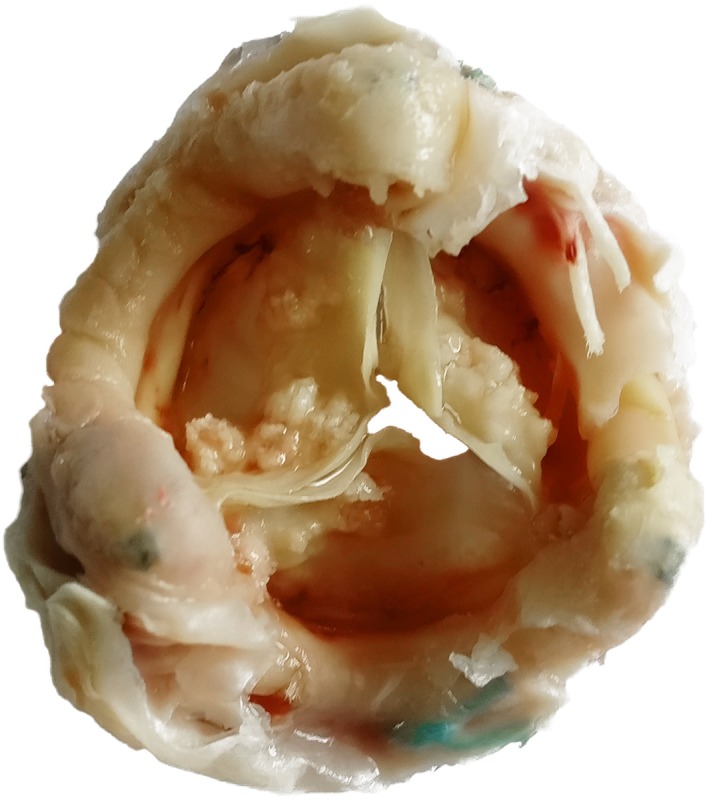
The explanted degenerative Epic bioprosthesis

**Figure 2:**
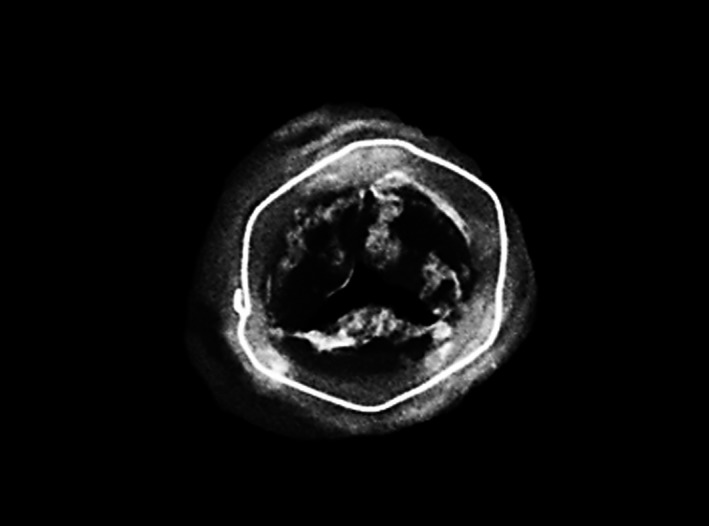
X‐ray examination of the explanted prosthesis showing acinar and linear calcifications in valve leaflets

During redo surgery, bioprosthetic valve leaflets were found to be sclerotic and unyielding, with multiple calcification nodules present on their aortic aspects (Figure 1). The bioprosthetic valve was replaced with a size 21 mm St. Jude Medical mechanical prosthesis, and X‐ray examination of the explanted prosthesis showed acinar and linear calcifications in valve leaflets (Figure 2). Postoperative recovery was uneventful, and echocardiography upon discharge demonstrated satisfactory mechanical valve function, with a mean gradient of 19 mm Hg and an effective valve orifice area of 1.72 cm^2^.

## 3 COMMENT

The Epic stented porcine bioprosthetic aortic valve has been associated with low percentages of structural failure.^1^ Beside the present case, only one prior report of early degeneration of the Epic valve appears in the medical literature.^2^ The primary pathology accounting for bioprosthetic valve failure is leaflet calcification and stiffening, resulting in valve degeneration. A variety of factors is known to induce leaflet calcification, mainly high serum calcium levels, vitamin D and calcium supplementations, chronic renal failure, and hemodialysis.^3^ Glutaraldehyde pretreatment has also been implicated in bioprosthetic valve degeneration. A reaction of membrane‐attached phosphorus with calcium‐loaded extracellular fluid can form phosphate‐calcium mineral deposits and could possibly initiate leaflet calcification.^4^ Another hypothesis is that glutaraldehyde fixation may not eliminate the antigenicity of bioprosthetic tissues fully, and that xenograft rejection and consequential calcification may be a reason for bioprosthetic valve failure.^5^ Leaflet calcific degeneration tends to progress slowly in old patients but is noticeably accelerated in younger subjects, and the process underlying this effect of age is still uncertain.^4^

None of the aforementioned risk factors for leaflet calcification were present in our patient; hence, early degeneration of this Epic valve under seemingly benign settings remains unaccountable. Since transcatheter aortic valve replacement (TAVR) is still not available in our country, we felt it was prudent to replace the degenerate valve, and we elected to use a mechanical prosthesis to preclude a second bioprosthetic valve failure, even though other physicians may contest this notion.

Even though early bioprosthetic valve failure remains infrequent, reporting such incidents is warranted to help recognize the mechanisms underlying early degeneration of bioprosthetic heart valves.

## CONFLICT OF INTEREST

All authors have declared that no conflict of interest exists.

## AUTHOR CONTRIBUTIONS

Mohammad Bashar Izzat, Nada Sabbagh, and Hazem Aljasem: involved in conception and acquisition of data, drafted the manuscript and revised it critically, and gave final approval of the version to be published.

## ETHICAL APPROVAL

All procedures performed in this study were in accordance with the ethical standards of the Damascus University Research Ethics Committee and with the 1964 Helsinki declaration and its later amendments.

## INFORMED CONSENT

Informed consent was obtained from all individual participants included in the study.

## References

[1] Jamieson WR , Lewis CT , Sakwa MP , et al. St Jude Medical Epic porcine bioprosthesis: results of the regulatory evaluation. J Thorac Cardiovasc Surg. 2011;141:1449‐1454. 21277603 10.1016/j.jtcvs.2010.05.055

[2] Wiedemann D , Bonaros N , Laufer G , Kocher A . Aortic bioprosthetic valve deterioration 8 months after implantation. Ann Thorac Surg. 2010;89:277‐279. 20103255 10.1016/j.athoracsur.2009.06.081

[3] Takano T , Terasaki T , Wada Y , et al. Early bioprosthetic valve calcification with alfacalcidol supplementation. J Cardiothorac Surg. 2013;8:11. 23324404 10.1186/1749-8090-8-11 PMC3554442

[4] Izutani H , Shibukawa T , Kawamoto J , Mochiduki S , Nishikawa D . Early aortic bioprosthetic valve deterioration in an octogenarian. Ann Thorac Surg. 2008;1369:1369‐1371. 10.1016/j.athoracsur.2008.03.064 18805202

[5] Manji RA , Zhu LF , Nijjar NK , et al. Glutaraldehyde‐fixed bioprosthetic heart valve conduits calcify and fail from xenograft rejection. Circulation. 2006;114:318‐327. 16831988 10.1161/CIRCULATIONAHA.105.549311

